# Functionalization of a versatile fluorescent sensor for detecting protease activity and temporally gated opioid sensing†

**DOI:** 10.1039/d4cb00276h

**Published:** 2025-02-18

**Authors:** Jennifer Sescil, Hailey Fiel, Steven M. Havens, Emma Fu, Xingyu Li, Kayla E. Kroning, Isabel Solowiej, Peng Li, Wenjing Wang

**Affiliations:** a Department of Chemistry, University of Michigan Ann Arbor MI 48109 USA wenjwang@umich.edu; b Life Sciences Institute, University of Michigan Ann Arbor MI 48109 USA; c Neuroscience Graduate Program, University of Michigan Ann Arbor MI 48109 USA; d Department of Biologic and Materials Sciences & Prosthodontics, University of Michigan Ann Arbor MI 48109 USA; e Department of Molecular and Integrative Physiology, University of Michigan Ann Arbor MI 48109 USA; f Program in Chemical Biology, University of Michigan Ann Arbor MI 48109 USA

## Abstract

Genetically encoded fluorescent sensors have been widely applied to detect cell signaling molecules and events. We previously designed a fluorescent sensor motif suitable for detecting protease activity and opioids. In this manuscript, we demonstrated the motif's first use for reporting on protease activity in animal models, demonstrating a high signal-to-background ratio of 29. We further functionalized this sensor motif to detect the activity of the coronavirus main protease, Mpro, and demonstrated its utility in characterizing an Mpro inhibitor. The Mpro sensor will facilitate the study of coronaviral activity in cell cultures and potentially in animal models. Additionally, we developed an innovative method for engineering a protease-based time-gating mechanism using this versatile sensor motif, allowing the temporally controlled detection of opioids. This time-gating strategy for detecting opioids can be generalized to other similar sensors, enabling detection of G protein-coupled receptor ligands with improved temporal resolution.

## Abstract 2

Genetically encoded fluorescent sensors have been widely applied to detect cell signaling molecules and events.

## Graphical abstract

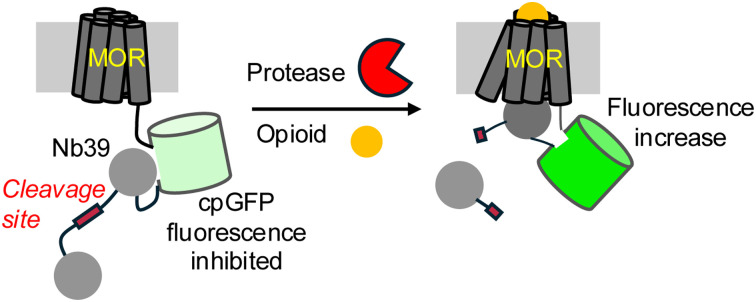

## Introduction

Genetically encoded sensors can read out cellular processes with high spatial resolution and cell-type specificity. Integrator reporters, so named because they integrate signal over time as opposed to a transient real-time readout, can permanently mark cells of interest with a fluorescent label for further analysis. Various genetically encoded integration sensors have been designed with different forms of fluorescent readout to detect G protein-coupled receptor (GPCR) ligands and protease activity across large volumes. These integrators can be classified as multiple- or single- component systems. Multiple- component integrators include TANGO,^1^ iTANGO,^2^ SPARK,^3,4^ CLAPon^5^ and split GFP.^6,7^ Single-component integrators include FlipGFP^8,9^ and the **S**ingle-chain **P**rotein-based **O**pioid **T**ransmission **I**ndicator **T**ool (SPOTIT) series of sensors.^10–13^ Multiple component integrator systems have the advantages of providing versatile readout and signal amplifications. However, they are limited by expression level differences between each component, which can affect the signal-to-background ratio (SBR) and therefore the robustness of each tool. Single-component integrator systems can address the high dependence on protein expression levels of multiple-component systems and provide more robust reporter systems.

Among the single-component reporters, the SPOTIT series of sensors show high versatility and functionalization. These sensors have proved effective for detecting both GPCR and protease activity. The SPOTIT integrator series include SPOTIT for detecting opioid molecules;^12,13^ SPOTon for detecting protease activity, protein–protein interactions and opioid molecules with a temporal gating;^11^ and SPOTall for detecting other GPCRs ligands, including those of the beta 2-adrenergic receptor, dopamine receptor D1 and cholinergic receptor muscarinic 2.^10^ These integrators all leverage a sensor motif composed of a circularly permuted green fluorescent protein (cpGFP) and the nanobody Nb39, which can intramolecularly interact with cpGFP and inhibit its fluorophore maturation. When Nb39 is removed *via* competitive binding, steric hindrance, or protease cleavage, cpGFP's fluorophore can mature and generate a permanent green fluorescence.^10^

The SPOTIT series has demonstrated its versatility and high potential for functionality but there are several areas that have not been addressed by these sensors. First, the SPOTon protease sensor has not been demonstrated in the mammalian brain. Second, the protease sensing has not been functionalized to meet the need for sensing diverse proteases important in viral infections, particularly the coronavirus main protease. Third, the SPOTIT sensors for reading out the presence of GPCR ligands, such as opioids, lack minimally perturbative time gating for highly sensitive detection. This manuscript thus focuses on addressing each of these three unmet needs through further functionalization of the SPOTIT series of sensors.

In this work, we characterized SPOTon's first use in animal models and demonstrated SPOTon-based detection of hepatitis C virus protease (HCVp) activity in the mouse brain. HCVp cleaves viral polyproteins into active proteins that infect cells.^14^ We further functionalized the SPOTon sensor by optimizing the protease detection motif to read out the activity of the coronavirus main protease, Mpro. Mpro cleaves the polyprotein encoded by the coronavirus genome into functional proteins that can infect healthy cells, resulting in the disease SARS-CoV-2.^15^ This optimized sensor, called Mpro-SPOTon, provides highly sensitive readout of Mpro activity that has the potential to be used in mammalian models. We further demonstrated Mpro-SPOTon's utility *via* a proof-of-principle analysis of an Mpro inhibitor.^16^ It showed potential for Mpro protease inhibitor screening and characterization, which are valuable for the development of coronavirus protease inhibitors as potential therapeutics.^15^

We also used the cpGFP-Nb39 motif to create a new tool allowing a user-defined time window for recording GPCR ligands, based on SPOTIT. While SPOTIT can sensitively and robustly detect GPCR ligands, the tool has poor temporal resolution, resulting in a gap in our ability to define when these GPCR ligands are released. The new tool, **MA**turation-based **P**rotease-gated **I**ndicator **T**ool (MAPIT), relies on the small molecule doxycycline-induced expression of a protease that can activate the integrator, enabling it to begin accumulating signal. Notably, MAPIT's time gating preserves the advantages of having a sensor on a single-chain. Additionally, using doxycycline for the time gating results in minimal interference with biological processes. As a small molecule-based time gating, instead of light-based systems, doxycycline can also diffuse through large volumes and have enhanced tissue penetration, which is important for detecting neurotransmitters that often travel far from their release site. We expect this time-gated GPCR ligand reporter to be expandable to the SPOTall system to detect other GPCR ligands besides opioids. This tool would potentially allow for the first time-gated recording of GPCR ligands across a large volume at high spatial resolution.

## Results

### Designing and characterizing the SPOTon sensor for protease detection

Tracking protease activity is important for studying many cellular processes. While some cleavage events are required for proper cell signaling, unregulated protease activity has been linked to cancer. Additionally, protease activity plays a crucial role viral infection.^17^ Detection of protease activity, especially by a generalizable tool that can be compatible with many protease types, is crucial for studying a variety of biological processes. As a genetically encoded protease sensor, SPOTon would enable detection in animal models across the organism with enhanced spatial resolution and cell type analysis. Analyzing protease activity across different cell types can reveal the specific cells involved in crucial cellular processes, especially during viral infections. Therefore, it is advantageous to have genetically encoded tools that can track protease activity with high sensitivity.

**Fig. 1:**
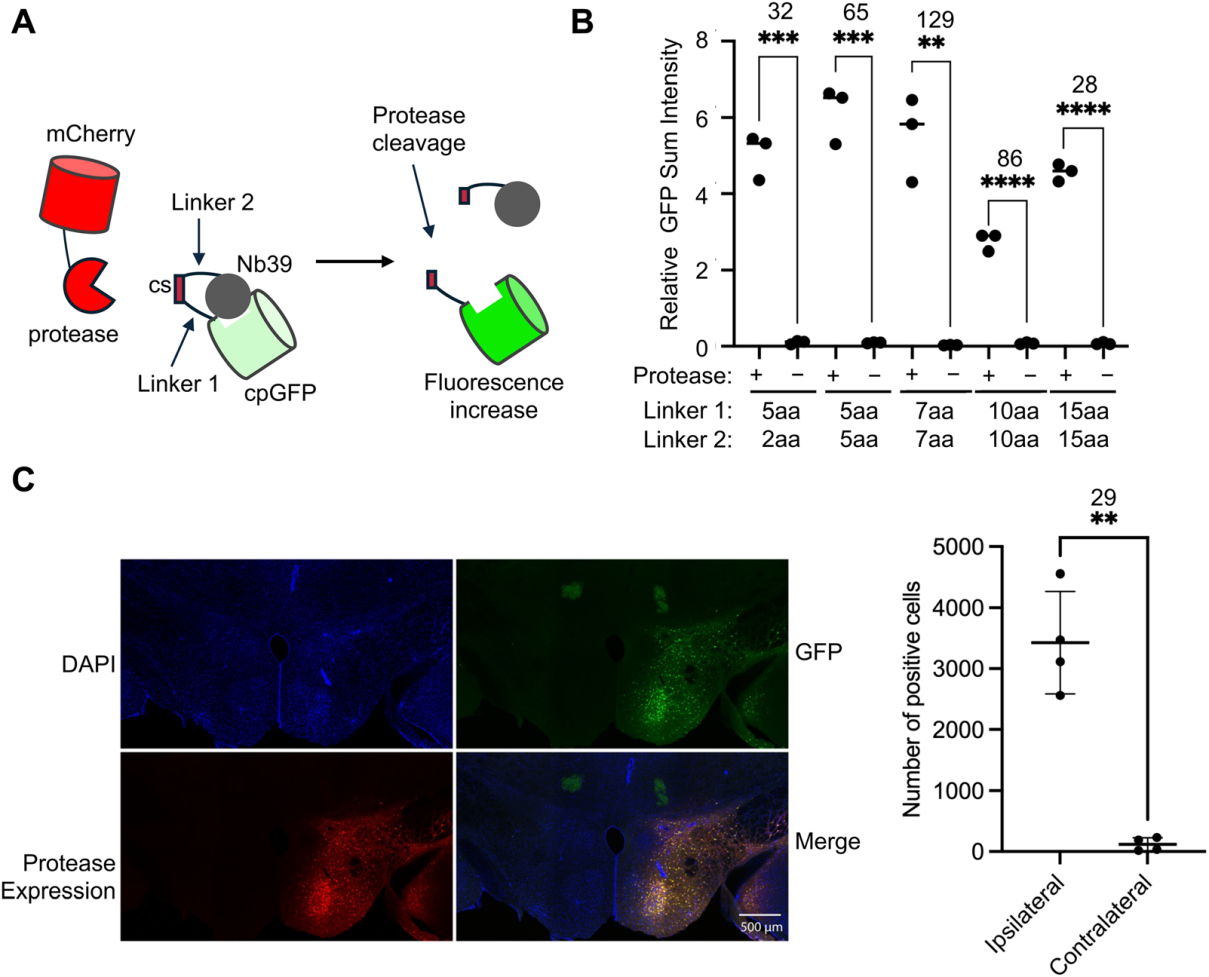
Characterization of SPOTon protease sensor. (A) Schematic of the SPOTon sensor. cs, cleavage site. (B) Testing different lengths of linkers around the hepatitis C virus cleavage site (HCVcs) in HEK293T cells. (C) Mammalian brain data of HCVp SPOTon. For (B) and (C) the thick horizontal bar is the mean value of three technical replicates (*n* = 3 and 4 for (B) and (C), respectively). The error bars represent SEM. The number above the stars is SBR between the two conditions and the stars indicate statistical significance. ****P < 0.0001, ***P < 0.001. **P < 0.01 Significance was calculated using an unpaired, two-tailed Student's *t* test for (B) and paired, two-tailed Student's *t* test for (C).

SPOTon provides a versatile single-component integrator system for detecting protease cleavage activity and was previously demonstrated with the tobacco etch virus (TEV) and hepatitis C virus (HCV) proteases.^11^ In SPOTon, the cpGFP is attached to Nb39 with a linker that includes a protease cleavage site (Fig. 1A). In the absence of protease, the cpGFP fluorophore maturation is inhibited by Nb39. Protease cleavage results in the removal of Nb39 from cpGFP, leading to fluorescence increase. Although the cpGFP-Nb39 motif is robust in SPOTon, we needed to ensure that this motif would be versatile enough to accommodate many types of proteases, which have cleavage sites that can greatly vary in length. To characterize SPOTon's dependence on linker length and to determine if longer linkers reduce the interaction between Nb39 and cpGFP in the absence of protease cleavage, we fused the cpGFP to the Nb39 *via* an HCVp cleavage site (HCVcs) with linkers of different lengths on either side (Fig. 1B and Fig. S1, ESI†). We characterized the SPOTon fluorescent signal with and without the HCV protease (HCVp). Even with the longest linkers tested, using 15 amino acids on either side of the cleavage site, the SPOTon sensor still produced a robust signal-to-background ratio (SBR) of 28. This shows that SPOTon is functional with long linker lengths, and therefore, even a long protease cleavage site can be tolerated and would not affect Nb39 inhibition of cpGFP fluorophore maturation in the absence of protease cleavage.

Next, we tested the suitability of SPOTon in mouse models, using HCVp-SPOTon. A concentrated AAV of mixed 1/2 serotype encoding the SPOTon sensor was injected into both the ipsilateral and contralateral sides of the mouse brain, with additional HCVp-encoding AAV co-injected on the ipsilateral side. In this setup, the contralateral side of the brain would show the background readout of the sensor in the absence of protease activity, and the ipsilateral side of the brain would show the sensor activation in the presence of protease activity. Seven days after the viral injection, the brain tissues were harvested for fluorescence analysis. The number of cells with green sensor fluorescence activation was compared between the contralateral side and the ipsilateral sides of the brain slices. Notably, we saw a high SBR of 29, indicating the SPOTon sensor is robust for use in mouse brains (Fig. 1C).

### SPOTon sensor for coronavirus protease detection

**Fig. 2:**
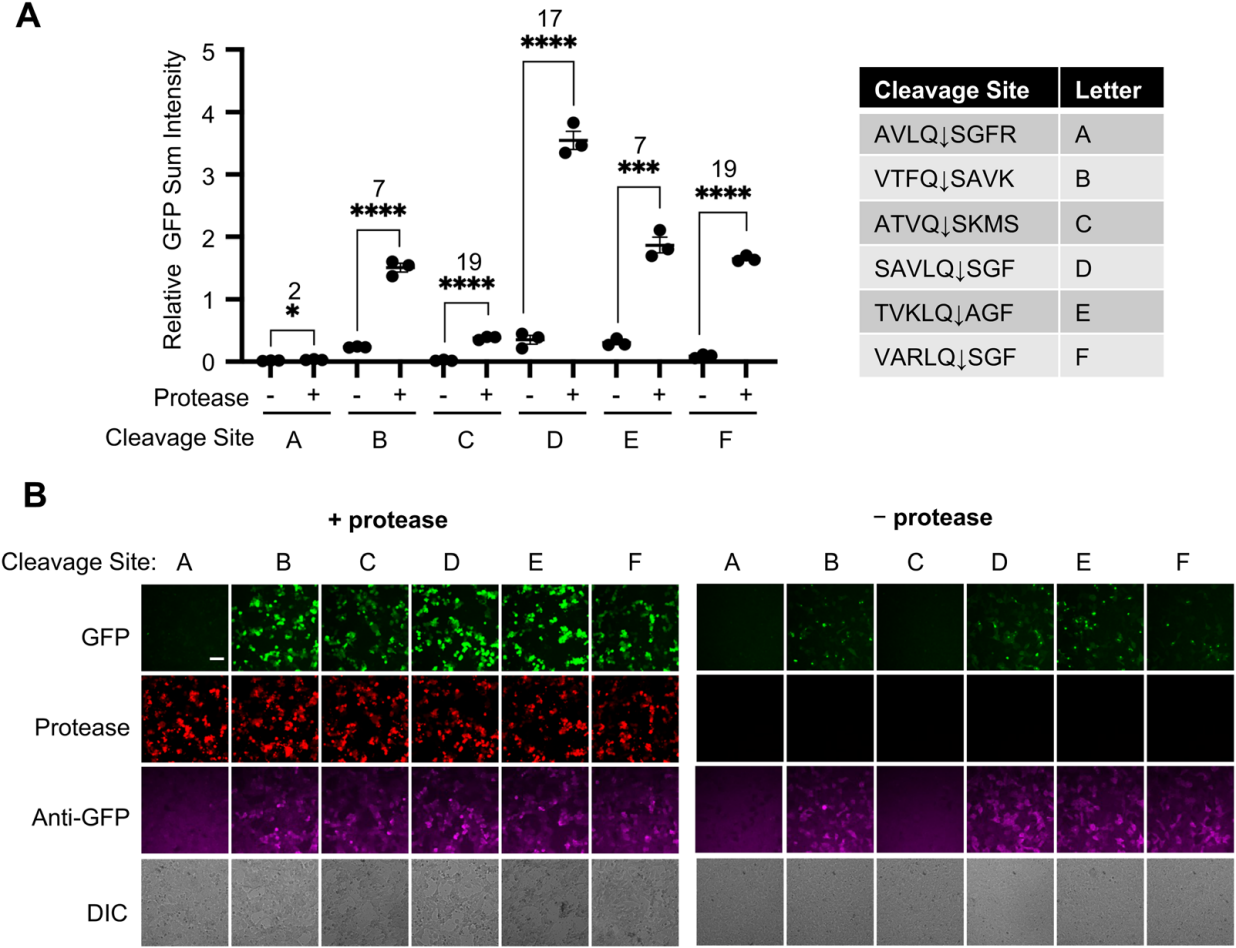
Development of a SPOTon sensor for coronavirus main protease (Mpro) in HEK cell culture. (A) Testing SPOTon using different main protease cleavage site (Mprocs), with and without Mpro. (B) Representative fluorescence images of (A). For (A) the thick horizontal bar is the mean value of three technical replicates (*n* = 3). The error bars represent SEM. The number above the stars is SBR between the two conditions and the stars indicate statistical significance. ****P < 0.0001, ***P < 0.001. ** *P* < 0.01, * *P* < 0.05. Significance was calculated using an unpaired, two-tailed Student's *t* test. Scale bar: 50 μm.

Due to the importance of proteases in viral infection, and the serious impact of SARS-CoV-2 pandemic on global public health systems, we further developed SPOTon for detecting Mpro, the main protease of the coronavirus. We tested six cleavage sites that had been characterized for their specificity for Mpro^18–21^ (Fig. 2). Among the cleavage sites tested, VARLQ↓SGF showed the best Mpro-dependent fluorescent activation, based on having the highest SBR, and a brighter overall signal compared to VTFQ↓SAVK, which had the same SBR. Even though AVLQ↓SGFR was previously found to exhibit high cleavage efficiency, we observed low activation signal in our sensor design.^18^ The immunofluorescence indicates low protein level using AVLQ↓SGFR, which could likely result from low protein stability for this sensor construct.

**Fig. 3:**
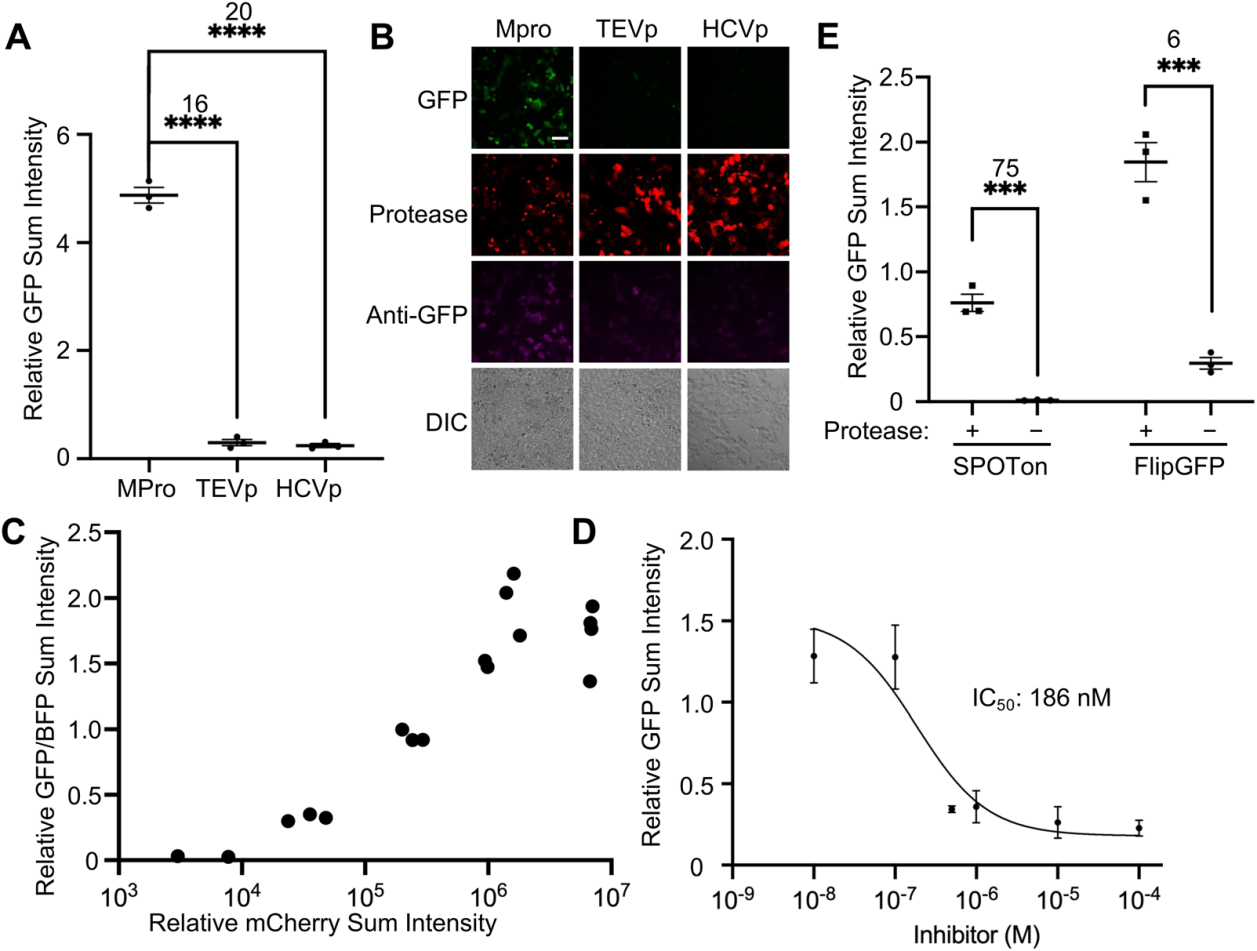
Characterization of a SPOTon sensor for coronavirus main protease (Mpro) in HEK 293T cell culture. (A) Testing the selectivity of the Mpro SPOTon sensor with Mpro, tobacco etch virus protease (TEVp), and hepatitis C virus protease (HCVp). (B) Representative fluorescence images for (A). Scale bar: 50 μm. (C) Testing the sensitivity of the Mpro sensor with varying amounts of the co-transfected Mpro. (D) Dose–response curve of the Mpro inhibitor GC376 with co-transfected Mpro and the Mpro sensor. For GC376, the IC_50_ range within 95% confidence is between 66 nM and 461 nM (*n* = 3). (E) Comparison of FlipGFP-Mpro and SPOTon-Mpro in HEK 293T cell culture. For (A) and (E) The thick horizontal bar is the mean value of three technical replicates (*n* = 3). The error bars represent SEM. The number above the stars is SBR between the two conditions and the stars indicate statistical significance. ****P < 0.0001, ***P < 0.001. Significance was calculated using an unpaired, two-tailed Student's *t* test.

We next characterized the specificity of SPOTon for detecting only the protease of interest, which is important for precise detection in cell cultures and live animals. We transfected HEK293T cells with the three SPOTon sensors for HCVp, TEV protease (TEVp), and Mpro, with their matched corresponding protease or a mismatched protease. As expected, for all three SPOTon sensors tested, the sensor is only activated when the protease and the corresponding cleavage site sensor are matched. When there is a mismatch in protease and sensor cleavage site, the sensor is not activated, resulting in a significantly lower fluorescence signal (Fig. 3A and B and Fig S2, S3, ESI†). This demonstrated that SPOTon sensors are specific for detecting the protease of interest, and that the optimized Mpro-SPOTon specifically detects Mpro activity.

We further characterized the sensitivity of Mpro-SPOTon to decreasing amounts of Mpro. To do so, we tested co-transfection of HEK293T cells with a constant amount of Mpro-SPOTon-p2a-BFP DNA and varying amounts of mCherry-p2a-Mpro DNA. When quantifying the sensor signal, we used a blue fluorescent protein (BFP) as an expression marker for Mpro-SPOTon and mCherry as an expression marker for Mpro (Fig. 3C and Fig S4, ESI†).

We chose to quantify using the BFP signal rather than the anti-GFP signal due to findings from the initial characterizations of SPOTon, which showed that SPOTon protein levels are lower without co-expression of the matching protease compared to levels with a matching protease.^11^ This result suggested that the SPOTon sensor is unstable in its inactive state, where fusion to Nb39 may destabilize the protein and cause faster degradation Consequently, the background signal of SPOTon without protease is low, which would provide higher SBRs for SPOTon and SPOTIT-related sensors.^11^ It is important to note that we quantified based on the BFP instead of using anti-GFP staining because the cpGFP in the sensor itself degrades when not activated. The BFP evades degradation because it is on a different protein chain. The Mpro-SPOTon sensor was found to be highly sensitive, detecting Mpro activity with an SBR of 11 at even the lowest amount of Mpro tested: 2 ng of Mpro DNA transfected per well of a 48-well plate (Fig S4, ESI†). Higher Mpro expression led to higher Mpro-SPOTon activation (Fig. 3C).

We also tested Mpro-SPOTon utility for screening protease inhibitors and characterizing protease inhibitor activity. As a proof-of-concept, we used the broad-spectrum antiviral GC376,^16^ a known Mpro inhibitor, to test with Mpro-SPOTon. If Mpro activity was inhibited, a decreased Mpro-SPOTon sensor signal would be observed. As an integration sensor that accumulates signal over time, the most sensitive Mpro-SPOTon version, containing VARLQ↓SGF, yielded saturated sensor signal even with reduced protease activity (data not shown). To better differentiate the reduced protease activity caused by inhibitors, we used a less sensitive version of the Mpro-SPOTon sensor, containing the cleavage site ATVQ↓SAVK. During transfection of HEK293T cells with Mpro and Mpro-SPOTon, increasing amounts of GC376 were added and allowed to incubate for one day, enabling expression of the sensor. This titration with the Mpro-SPOTon sensor produced as an IC50 of approximately 186 nM, with 95% confidence that the IC50 is between 66 and 461 nM (Fig. 3D and Fig. S5, ESI†).

Lastly, we compared Mpro-SPOTon with an existing protease sensor based on FlipGFP. Since SPOTon tends to be degraded in the absence of protease,^11^ we expected SPOTon to have a decreased background compared to existing tools such as FlipGFP, and therefore a higher SBR. This was found to be the case for Mpro-SPOTon, which outperformed Mpro-FlipGFP with an SBR of 75 compared to Mpro-FlipGFP's SBR of 6 (Fig. 3E and Fig. S6, ESI†). We also demonstrated the improved SBR of the SPOTon sensor compared to FlipGFP for the TEVp (Fig. S7, ESI†).

### Designing MAPIT for time-gated detection of opioids

Due to the importance of opioids and other G protein-coupled receptor ligands in controlling biological processes, we wanted to have a sensor that could detect the release of these signaling molecules. The single-chain nature of SPOTIT prevents variation in signal due to the differing expression levels of multi-component sensors. However, the sensor lacks time gating, since the fluorophore maturation is irreversible, and it is difficult to define when the opioid signaling event occurred. A rapamycin-dependent split SPOTIT had been developed to address the need for time gating but involved a two-component system and suffered from a greatly reduced sensitivity for detecting opioids.^11^ Therefore, there is still a need for a new mechanism of time gating that can sensitively detect opioids and retain the advantages of the single-chain sensor.

**Fig. 4:**
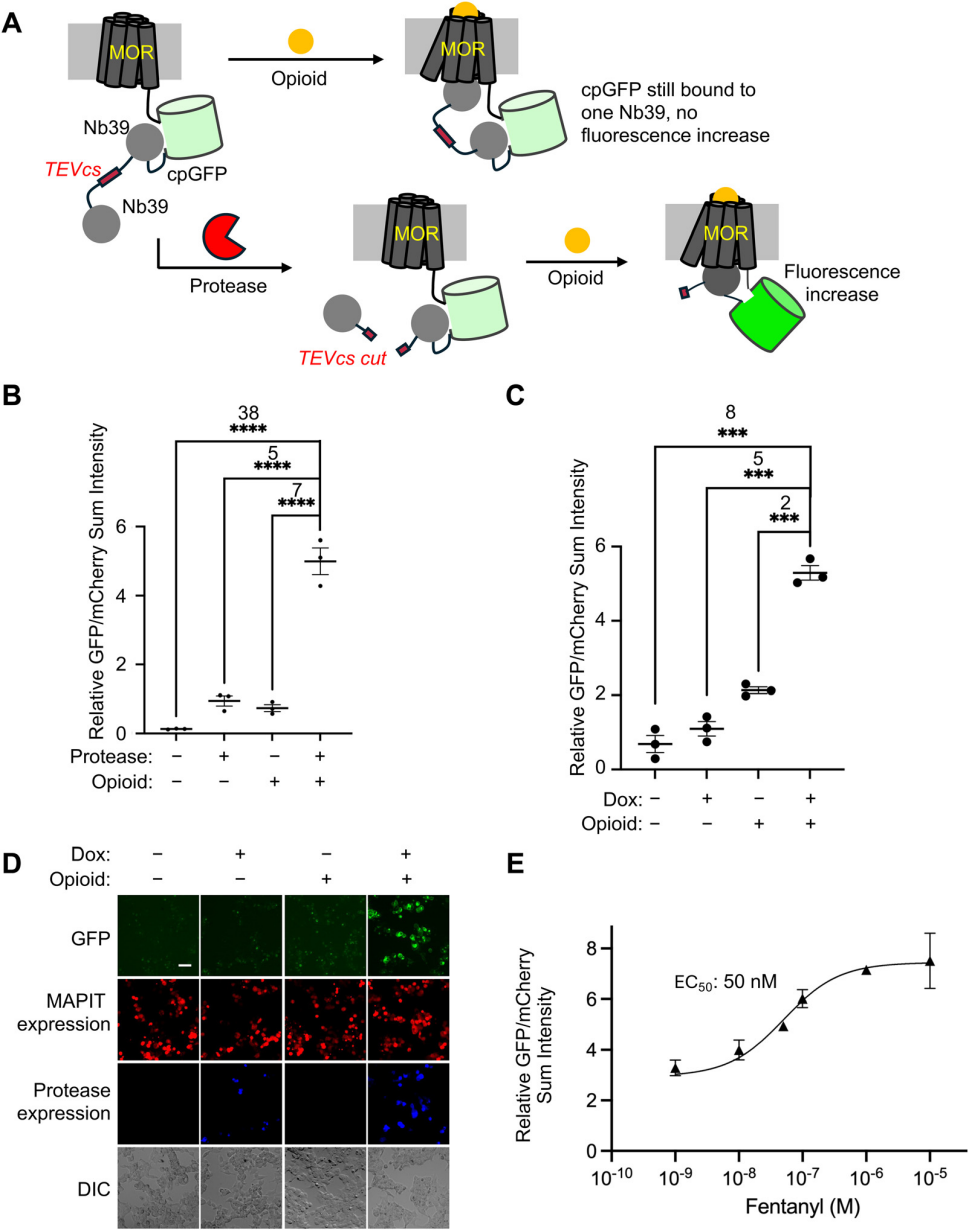
MAPIT provides a protease-based time-gating for an opioid sensor in HEK cell culture. (A) Schematic of MAPIT. (B) Testing MAPIT with and without co-transfected TEVp and 10 μM loperamide. (C) Testing the doxycycline-induced expression of TEVp with the MAPIT sensor, with and without twenty-four hours of doxycycline and 10 μM loperamide. (D) Representative fluorescence images for (C). Scale bar: 50 μm. (E) Dose–response curve of Dox-MAPIT sensor for fentanyl. For fentanyl, the EC_50_ range within 95% confidence is 19 to 123 nM. The mean is represented by each point in the plot (*n* = 3). For (B) and (C) the thick horizontal bar is the mean value of three technical replicates (*n* = 3). ****P < 0.0001, ***P < 0.001. The error bars represent SEM. The number above the stars is SBR between the two conditions and the stars indicate statistical significance. Significance was calculated using an unpaired, two-tailed student's *t* test.

To address this need, we engineered a new time-gated GPCR activity integrator by incorporating an additional Nb39 to SPOTIT *via* a protease cleavage site. This new reporter is called **MA**turation-based **P**rotease-gated **I**ndicator **T**ool (MAPIT). In MAPIT (Fig. 4A), the second Nb39 acts as an additional inhibitory motif for cpGFP's fluorophore maturation. As a result, in the presence of opioid, when one Nb39 is recruited to the single Nb39 binding pocket on the activated mu-opioid receptor (MOR), the cpGFP is still inhibited by the other Nb39, and therefore, its fluorophore maturation is still inhibited. When a protease cleavage event removes the second Nb39, MAPIT becomes similar to SPOTIT, and can start integrating opioid signal (Fig. 4A). In the design of MAPIT, we used a TEVp cleavage site (TEVcs) to fuse the second Nb39, so that TEVp activity initiates the MAPIT sensor opioid signal integration.

To test MAPIT, we transfected MAPIT with or without TEVp, and stimulated the cells with or without the MOR agonist, loperamide. Fig. 4B and Fig S8 (ESI†) shows that, as expected, MAPIT has high fluorescent signal only in the presence of both TEVp and loperamide. In the presence of TEVp, MAPIT shows an opioid-dependent SBR of 5, comparable to that of SPOTIT.^12,13^ Without TEVp, even with loperamide stimulation, low fluorescence was observed due to the continuous cpGFP fluorophore inhibition by the other Nb39, showing a protease-dependent SBR of 7.

Next, to create a user-defined time window of opioids signal recording, we incorporated designs to control the initiation of the protease-cleavage event. We chose to use small molecules for the time gating of the protease cleavage events, as small molecules can penetrate deep tissues and allow large volume recording of MAPIT. We tested two different small molecule-dependent designs for inducing protease activity: a rapamycin-dependent split protease design (Rap-MAPIT) (Fig S9, ESI†), and a doxycycline (Dox)-inducible expression of the protease design (Dox-MAPIT) (Fig. 4C and D).

In Rap-MAPIT, we introduced a rapamycin-dependent split TEVp to control the protease cleavage event. In this design, we used a highly active TEVp cleavage site of ENLYFQG and a split truncated TEVp that shows reduced background reconstitution before rapamycin addition.^22^

MAPIT and the rapamycin-dependent split TEVp constructs were co-transfected in HEK293T cells followed by stimulation under four conditions, with or without rapamycin and loperamide. Unfortunately, we did not observe any rapamycin-dependence (Fig S9, ESI†). This is most likely due to the background split TEVp reconstitution in the absence of rapamycin. Further optimization could potentially be achieved by tuning the TEVcs or further improving the split TEVp system.

In the Dox-MAPIT design, we introduced the Tet-On system^23,24^ to initiate the transcription and translation of TEVp only upon addition of Dox. In this design, we tested using a highly active TEVp cleavage site of ENLYFQA and the full length TEVp with the S153N point mutation for improved kinetics.^25^ The MAPIT sensor and the Tet-On-TEVp were co-transfected in HEK293T cells, followed by stimulation under four conditions, with or without Dox and opioid. As expected, stimulation with both Dox and opioids gave the best MAPIT activation signal, with a Dox-dependent SBR of 2 and an opioid-dependent SBR of 5 (Fig. 4C and D). The background signal without Dox but with opioid is most likely due to the leaky expression of TEVp using the Tet-On system in HEK cells. The Dox-dependence can potentially be further improved by using an even tighter Tet-On system or using a lower activity pair of TEVp and TEVp cleavage site.

In order to evaluate the doxycycline incubation time needed for Dox-MAPIT activation, we transfected Dox-MAPIT constructs in HEK293T cells. The following day, the cells were stimulated with doxycycline for six hours and then stimulated with fentanyl for an additional twenty-four hours. This is because the fluorophore maturation of the opioid sensor requires six hours to produce a noticeable change and twenty-four hours for a much more robust signal.^13^ Fig. S10 (ESI†) shows that six hours of doxycycline stimulation is sufficient for doxycycline-gated MAPIT activation.

We next characterized the sensitivity of Dox-MAPIT. In the previous time-gated split-SPOTIT design,^11^ the sensitivity of split-SPOTIT towards fentanyl is significantly reduced, with an EC_50_ of 850 nM^11^ compared to an EC_50_ of 30 nM for SPOTIT.^13^ This decrease is presumably due to the reduced efficiency of SPOTIT activation in a two-component system even when the split SPOTIT is brought together and reconstituted. To evaluate the sensitivity of Dox-MAPIT, we performed a fentanyl dose response curve for Dox-MAPIT in the presence of Dox. An EC_50_ of 50 nM was observed (Fig. 4E and Fig S11, ESI†), similar to that of SPOTIT and much more sensitive than that for the time-gated split-SPOTIT.

Overall, we have developed a sensitive and single-chain version of time-gating for the versatile SPOTIT sensor series using the minimally perturbative small molecule doxycycline. We expect this motif to be generalizable to SPOTall sensors to detect many kinds of GPCR ligands with time-gating across a whole organism.

## Discussion

In this study, we present the functionalization of a versatile fluorescent maturation motif for robust detection of different protease activity, including the coronavirus Mpro, which outperformed an existing FlipGFP-based protease sensor.^8,9^ For the first time, we demonstrated SPOTon's suitability for detecting protease activity in mouse brains with a high SBR of 29. Detecting specific protease activity would be a useful proxy for detecting cells that are vulnerable to specific viral infections. A genetically encoded integrator like SPOTon would be suitable for use in animal models across the organism with enhanced spatial resolution and cell type analysis. We have shown the protease sensor to be useful in different applications, including characterization of protease inhibitors, which have potential as antiviral therapeutics. Given its high brightness and SBR, Mpro-SPOTon would be an ideal candidate for testing coronavirus main protease activity in animal models. This green fluorescent protease sensor could potentially be used for multiplexed detection of protease activity along with the red SPOTon protease sensor.^10^

Additionally, we present a new time-gated opioid-integration reporter MAPIT using the same fluorescent maturation motif. This new design retains the advantages of a sensitive single-chain sensor of SPOTIT using the small molecule Dox with minimal perturbation of endogenous systems. Dox-MAPIT provides a similar sensitivity to fentanyl when compared to M-SPOTIT-2, unlike the previous rapamycin-gated split SPOTIT, which had decreased sensitivity.^11,13^ Additionally, this small molecule can penetrate deep tissues, potentially enabling the whole brain mapping of the opioids release. We expect this new time-gating strategy to be generalizable for the many kinds of GPCR ligands that can be detected in SPOTall,^10^ as well as for the red-SPOTIT sensor for multiplexed recording.

It is also important to note the limitations of MAPIT, which will likely require further screening of different TEVp and TEVcs pairs to improve the doxycycline-dependence and address the Tet-On system's leakiness. Using a lower affinity TEVcs or less active TEVp could reduce the amount of protease cleavage from leaky protease expression but must be screened to retain high activation in the presence of doxycycline. This improvement on the MAPIT sensor would facilitate, for the first time, the time-gated detection of GPCR ligands across large volumes in animal models with high spatial resolution.

## Author contributions

W. W., J. S. H. F. and K. K. conceptualized the project. All authors designed experiments. J. S. performed the cell culture experiments. K. E. K and H. F. designed the Mpro-SPOTon development and characterization. S. M. H. performed data analysis. E. F., H. F. and J. S. cloned the DNA constructs. I. S. and X. L. performed the animal experiments. P. L. and W. W. contributed new reagents/analytic tools. J.S. and W. W. wrote the initial manuscript draft and all authors edited and revised the manuscript.

## Ethical statement

All procedures followed the National Institutes of Health (NIH) guidelines and were approved by the Institutional Animal Care and Use Committee (IACUC) at the University of Michigan.

## Data availability

All the data supporting the findings of this study are available within the paper and its ESI.† All the DNA constructs used in this study are available upon request to the corresponding author.

## Conflicts of interest

There are no conflicts of interest to declare.

## Supplementary Material

CB-OLF-D4CB00276H-s001
