# Supplementary material for: Functionalization of a versatile fluorescent sensor for detecting protease activity and temporally gated opioid sensing
Source: RSC Chem Biol. 2025 Feb 18;6(4):555–62. doi: 10.1039/d4cb00276h (PMC11835013; doi:10.1039/d4cb00276h)
Supplement: CB-006-D4CB00276H-s001 [file CB-006-D4CB00276H-s001.pdf]

Supplementary Information (SI) for RSC Chemical Biology.
This journal is © The Royal Society of Chemistry 2025

Supplementary Information

Plasmids and Cloning.

Q5 PCR amplification, NEB restriction enzyme digest and Gibson assembly were used to produce the DNA plasmids. Except for the doxycycline-dependent protease expressed using the TETon system, all DNA constructs were cloned into an adeno-associated viral vector with ampicillin resistance, under a CAG promoter. The AAV vector that was used to express the doxycycline-dependent protease in our study was constructed and packaged by VectorBuilder. The vector ID is VB010000-0053bwh, which can be used to retrieve detailed information about the vector on vectorbuilder.com. XL-1 blue competent cells were transformed with these plasmids using heat-shock.

Cell Culture and Transfection

HEK293T cells (ATCC: CRL-3216) were cultured in 1:1 MEM:DMEM media (Fisher) with 10% v/v fetal bovine serum (Biowest), 20 mM HEPES (ThermoFisher) and 1% (v/v) penicillin-streptomycin (Gibco). The cells were cultured at 37 °C under 5% CO_2.

For transfections, 48 well plates were pre-treated with 200 µL or 40 µg mL^{-1} human fibronectin (Millipore Sigma) for 10 min at 37 °C, and then the fibronectin was aspirated. 100 ng of each DNA construct was incubated for 10 minutes at room temperature with 10 uL of DMEM and 1 µL of PEI per well of a 48 well plate, with the exception of Supplementary Figure 4 and Figures 3C, 4C and 4D. In Supplementary Figure 4 and Figure 3C, 100 ng of Mpro-SPOTon was co-transfected with varying amounts of Mpro, which are identified in Supplementary Figure 4. In Figures 4C and 4D, 50 ng of the doxycycline-inducible protease was co-transfected with 100 ng of the MAPIT sensor. Per well of a 48 well plate, 100 µL of complete media and 200 µL of an 80% confluent HEK293T cell in suspension were added to the transfection mixture and mixed by pipetting. 310 µL of this mixture was then plated in each well of a 48 well plate.

HEK293T Cell Stimulation

For Figure 3D and Supplementary Figure 5, the varying concentrations of protease inhibitor GC376 (Aobious Inc) were diluted in complete media to a final volume of 100 µL. This 100 µL of GC376 was added to the cells at the time of transfection. For Figure 4 and Supplementary Figure 8, 9 and 10 and 11, the doxycycline, loperamide or fentanyl was added 20-24 hours post transfection after being diluted in complete media. The doxycycline was at a concentration of 0.5 µg mL^{-1}. In Supplementary Figure 11, the doxycycline was removed by washing twice with complete media before the addition of fentanyl or media. The loperamide was at a final concentration of 10 µM, and the fentanyl concentration was varied to produce a dose-response curve. These stimulations were always an addition of 100 µL of media containing the drug(s).

Fixation and Immunostaining

For the SPOTon experiments, fixation occurred 20-24 hours after transfection, and for MAPIT experiments, fixation occurred 20-24 hours after stimulation. The supernatant was aspirated and 200 µL of 4% paraformaldehyde (PFA, Electron

Microscopy Science, #15713) was added per well of a 48 well plate. The plate was incubated at room temperature for 15 minutes. The PFA was aspirated and the wells were washed twice with phosphate-buffered saline (PBS). The cells were then permeabilized with 200 µL of ice-cold methanol for 5 minutes, followed by two more washes with PBS. Then, 100 µL of primary antibody was added per well and the plate was rocked for 30 minutes. Following two washes, 100 µL of secondary antibody was added per well and the plate was rocked for 30 minutes. All antibodies were diluted to 1 µg L^{-1} in 1% bovine serum albumin (DOT) in PBS. The primary antibody used was chicken anti-GFP (Abcam) and the secondary antibody used was AlexaFluor 647 goat anti-chicken IgG (Life technologies). Following immunostaining, the cells were washed twice with PBS and fixed again with 200 µL of 4% paraformaldehyde for 15 minutes at room temperature. The PFA was aspirated, and the cells were washed twice with PBS and then imaged in pH 11 CAPS buffer.

Confocal Microscopy

A Nikon inverted confocal microscope with a 20x air objected was used for confocal imaging. The microscope was outfitted with a Yokogawa CSU-X1 5000RPM spinning disk confocal head and Ti2-ND-P perfect focus system 4, a compact 4-line laser source: 405 nm (100 mW) 488 nm (100 mW), 561 nm (100mW), and 640-nm (75 mW) lasers. The laser excitation and emission filters used for the various fluorophores were: DAPI/BFP (405 nm excitation; 455/50 emission); cpGFP (488 nm excitation; 525/36 emission); mCherry (568 nm excitation; 605/52 emission); and AlexaFluor-647 (647 nm excitation; 705/72 emission). The 10x objective was used for mouse brain-slice imaging. The 20x objective was used for the HEK293T cell experiments. All of the images were collected using Nikon NIS-Elements hardware control.

Analysis of HEK293T cell images.

NIS-Elements General Analysis 3 software was used to analyze all images. For each experiment, the fluorescence background intensity was calculated by taking the mean intensity of an area devoid of cells for each emission wavelength. A threshold was used at twice the background intensity to select cell areas with real fluorescence, and the mean intensity values and total object areas were determined. The sum intensity of each image was calculated by subtracting the background intensity from the mean intensity, before multiplying by the total object area. For ratiometric experiments, the sum intensity of the signal channel was then divided by the sum intensity of the expression channel. The sum intensity values, or ratiometric sum intensity values, of all images within a well were then averaged and each technical replicate was plotted as one point in the graph. Graphs were plotted with all intensity values divided by the highest order of magnitude in the experiment, to generate relative fluorescence intensity values. All images were included in analysis unless the image contained autofluorescence artifacts or had lower than 50% cell density. The Prism GraphPad software was used to plot all data and calculate statistical significance through paired and unpaired two-sided Student's *t*-tests.

Virus Preparation and Animal Experiments

The AAV1/2 virus for use in animal experiments was produced and concentrated using the same protocol that Shen et al. described. The stereotactic injection procedure closely followed established protocols (Li et al. 2020). Adult C57BL/6 mice of both sexes, aged 8–24 weeks, were used in this study. Mice were housed under a 12-hour light/dark cycle at 22 °C, with food and water available ad libitum.

In summary, adult mice were anesthetized using isoflurane (5% for induction, 1.5% for maintenance) and administered 5 mg kg^{-1} of carprofen. They were then positioned in a stereotactic apparatus, ensuring body temperature maintenance within the range of 35–37 °C. 500 nL of concentrated AAV encoding HCVp or HCVp and HCVp-SPOTon were precisely injected into the lateral hypothalamic area (±0.95 mm lateral to midline, −1.40 mm posterior, and −5.25 mm ventral to bregma) at a controlled rate of 50 nL min^{-1}. Following injection, the pipette remained stationary within the brain for 10 minutes to allow pressure equalization and prevent a vacuum effect upon removal. Post-surgery, mice received subcutaneous injections of 1 mL saline and were allowed to recover. An additional subcutaneous dose of 5 mg kg^{-1} carprofen was administered the following day for pain management.

Seven days post-injection of the viral vectors, the animals were euthanized and underwent perfusion with PBS followed by 4% paraformaldehyde (PFA, Electron Microscopy Science, #15713). Brain tissues were carefully extracted and subsequently fixed overnight in 4% PFA, followed by cryoprotection in 30% sucrose for 48 hours at 4 °C. The fixed tissues were then embedded in optimum cutting temperature compound, sliced into 40 μm sections, and mounted onto glass slides. These tissue sections were stained with DAPI (1:10,000, Invitrogen, #D1306) for 10 minutes at room temperature, after which confocal images were captured using a Nikon A1 Confocal microscope.

Supplementary Figures

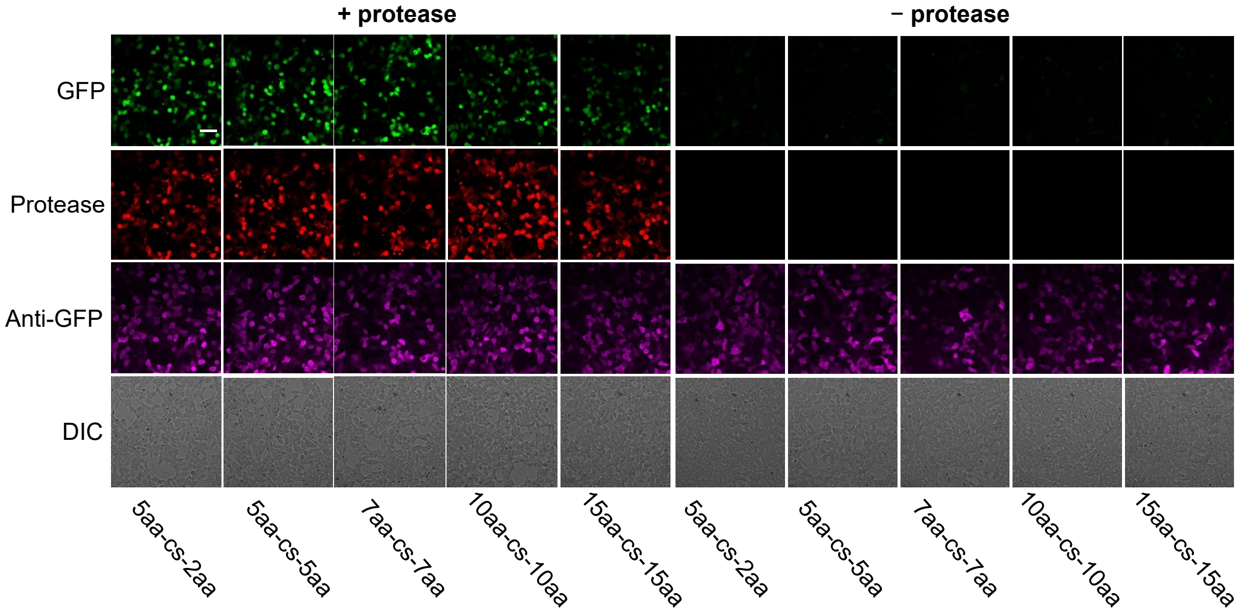

Supplementary Fig 1. Representative fluorescence images for Figure 1B. Scale bar: 50 μm.

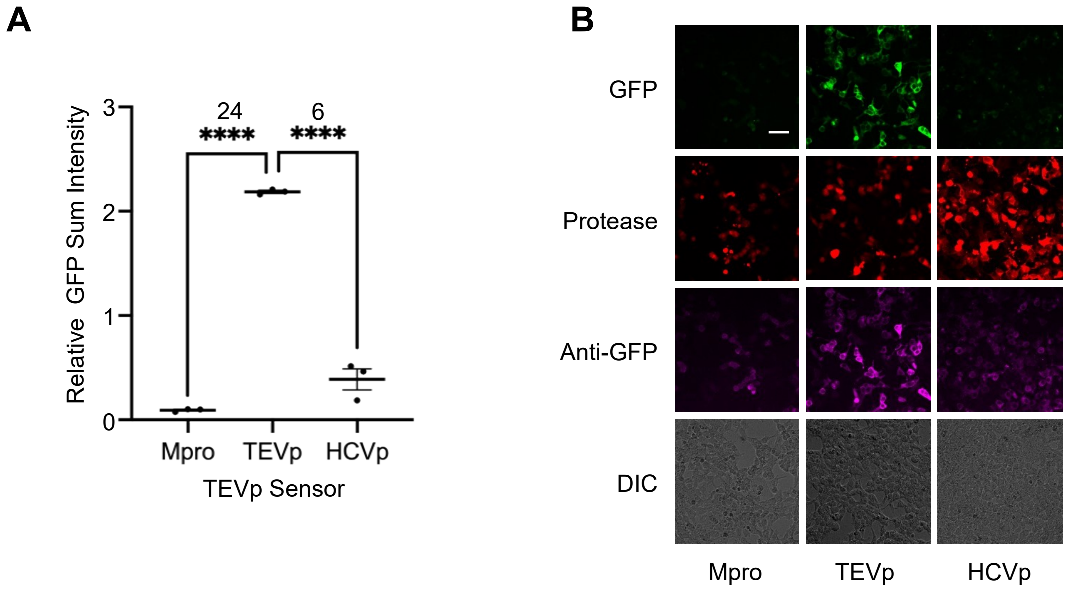

Supplementary Fig 2. A. Testing the selectivity of the tobacco etch virus protease (TEVp) sensor with TEVp, the coronavirus main protease (Mpro), and hepatitis C virus protease (HCVp) in HEK 293T cell culture. **B.** Representative fluorescence images of A. For A: The thick horizontal bar is the mean value of three technical replicates (n=3). The error bars represent SEM. The number above the stars is SBR between the two conditions and the stars indicate statistical significance. ****P < 0.0001Significance was calculated using an unpaired, two-tailed Student’s t test. Scale bar: 50 μm.

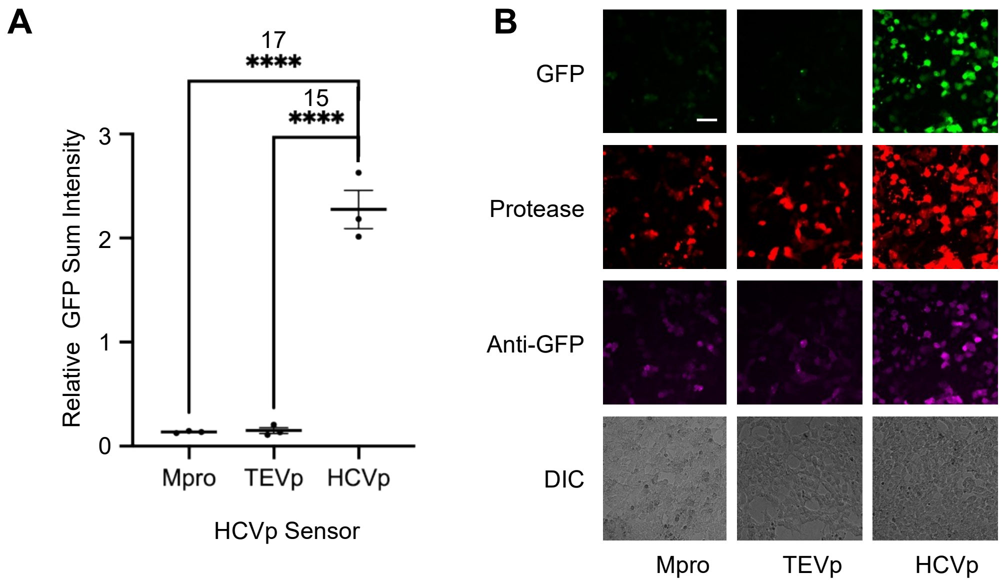

Supplementary Figure 3. A. Testing the selectivity of the hepatitis C virus protease (HCVp) sensor with HCVp, the coronavirus main protease (Mpro), and tobacco etch virus protease (TEVp) in HEK 293T cell culture. **B.** Representative fluorescence images of A. For A: The thick horizontal bar is the mean value of three technical replicates (n=3). The error bars represent SEM. The number above the stars is SBR between the two conditions and the stars indicate statistical significance. ****P < 0.0001, ***P < 0.001. Significance was calculated using an unpaired, two-tailed Student's t test. Scale bar: 50 μm.

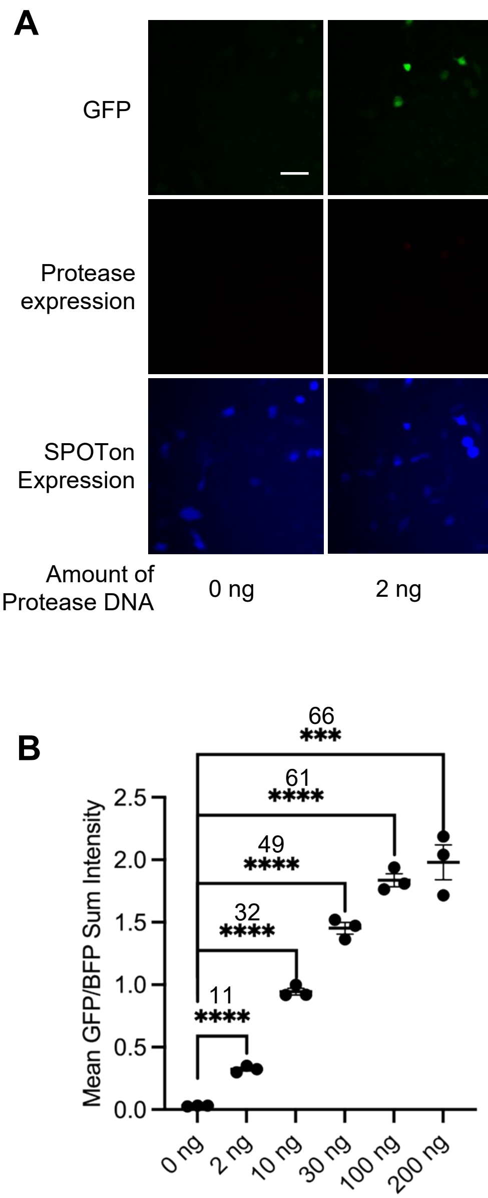

Supplementary Figure 4. A. Representative fluorescence images from Figure 3C, showing the amount of protease co-transfected in HEK 293T cells with 100 ng of Mpro SPOTon. Scale bar: 50 μm **B.** Testing the sensitivity of the Mpro sensor with varying amounts of the co-transfected Mpro DNA. The thick horizontal bar is the mean value of three technical replicates (n=3). The error bars represent SEM. The number above the stars is SBR between the two conditions and the stars indicate statistical significance. ****P < 0.0001, ***P < 0.001. Significance was calculated using an unpaired, two-tailed Student's t test.

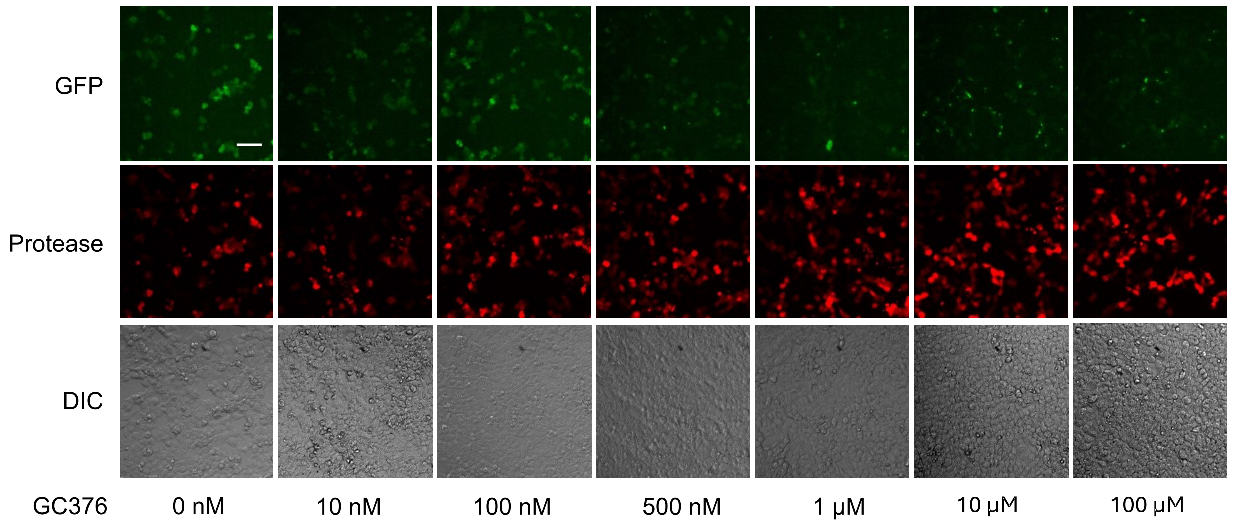

Supplementary Figure 5. Representative fluorescence images from Figure 3D, showing the amount of GC376 used to stimulate HEK293T cells co-transfected with the Mpro-SPOTon and Mpro. Scale bar: 50 μm.

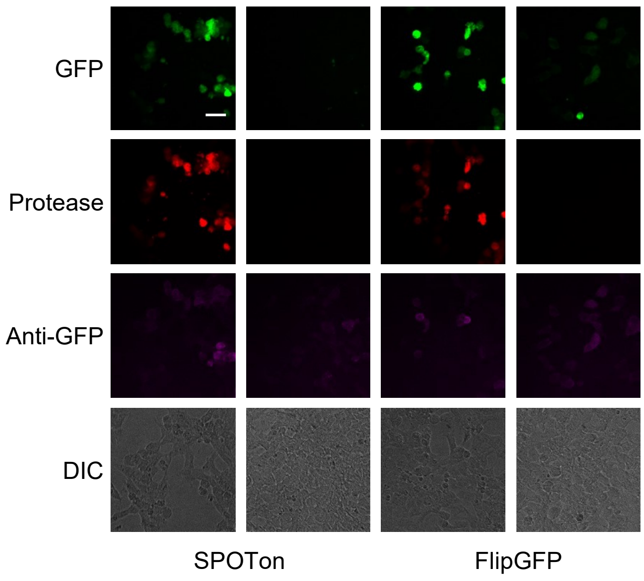

Supplementary Figure 6: Representative fluorescence images from comparison of FlipGFP-Mpro and SPOTon-Mpro in HEK 293T cell culture from Figure 3. Scale bar: 50 μm.

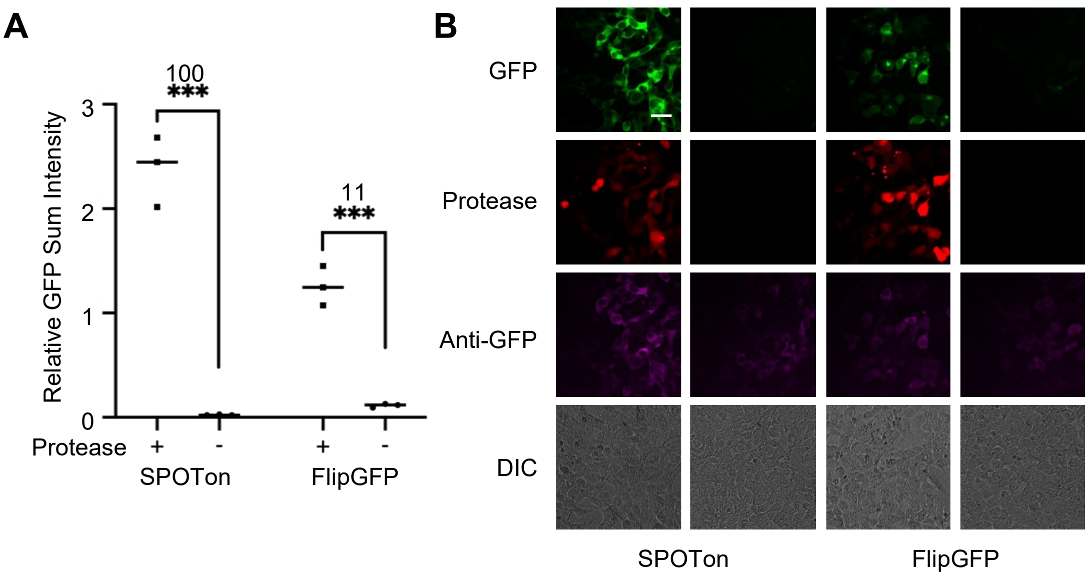

Supplementary Figure 7. A. Comparison of FlipGFP-TEVp and SPOTon-TEVp in HEK 293T cell culture. **B.** Representative fluorescence images from A. For A: The thick horizontal bar is the mean value of three technical replicates (n=3). The error bars represent SEM. The number above the stars is SBR between the two conditions and the stars indicate statistical significance. . ****P < 0.0001, ***P < 0.001. Significance was calculated using an unpaired, two-tailed Student's t test.

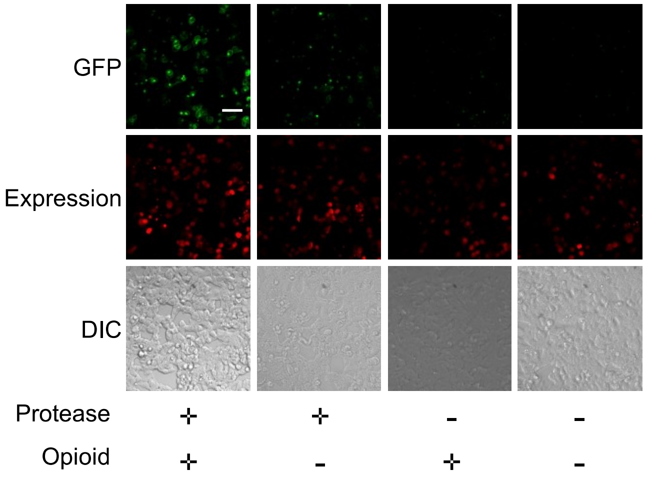

Supplementary Figure 8. Representative fluorescence images for Figure 4B. Scale bar: 50 μm.

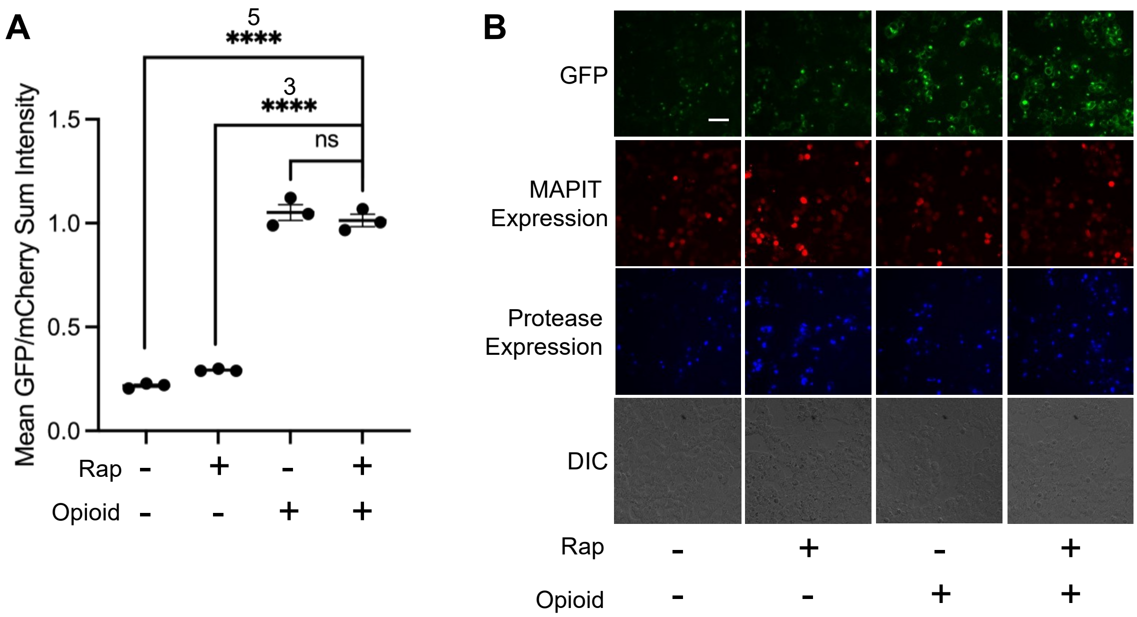

Supplementary Figure 9. A. Testing the rapamycin-induced expression of TEVp with the MAPIT sensor, with and without 10 μM loperamide and 1 μM rapamycin. **B**. Representative fluorescence images for A. The thick horizontal bar is the mean value of three technical replicates (n=3). The error bars represent SEM. The number above the stars is SBR between the two conditions and the stars indicate statistical significance. ****P < 0.0001, ns, no significance. Significance was calculated using an unpaired, two-tailed Student's t test. Scale bar: 50 μm.

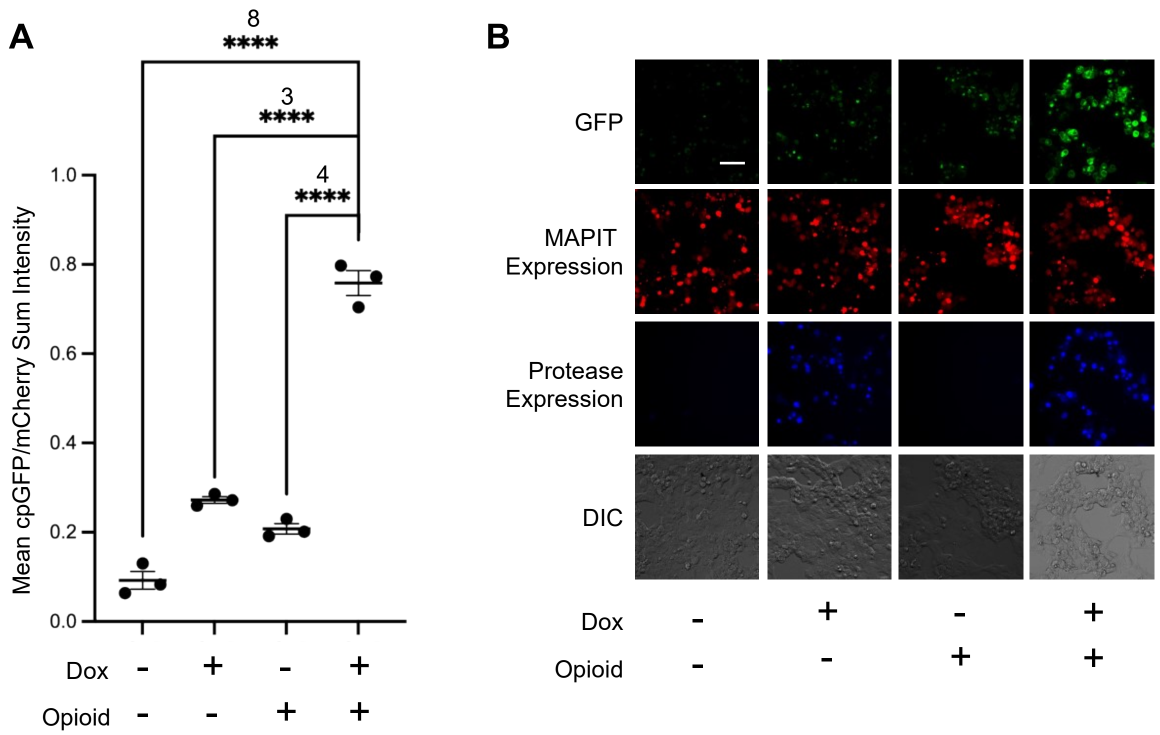

Supplementary Figure 10. A. Testing the doxycycline-induced expression of TEVp with the MAPIT sensor, with and without six hours of doxycycline exposure followed by with or without 10 μM fentanyl . **B**. Representative fluorescence images for A. The thick horizontal bar is the mean value of three technical replicates (n=3). The error bars represent SEM. The number above the stars is SBR between the two conditions and the stars indicate statistical significance. ****P < 0.0001, ns, no significance. Significance was calculated using an unpaired, two-tailed Student's t test. Scale bar: 50 μm.

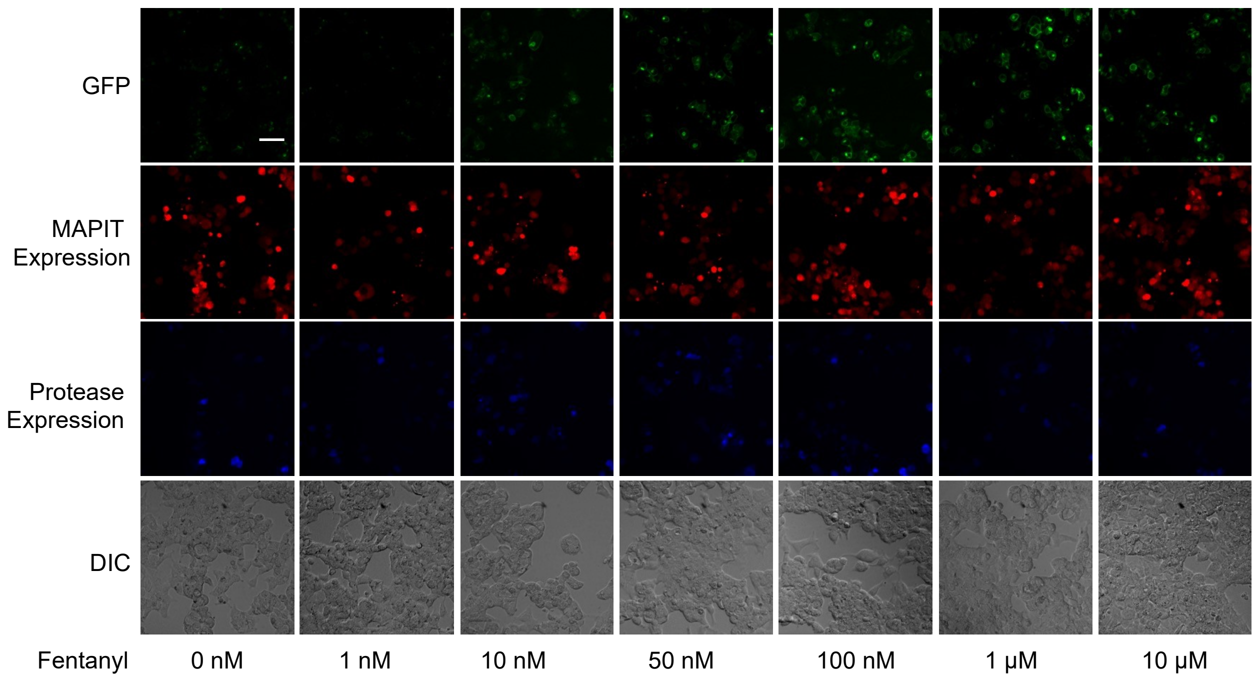

Supplementary Figure 11: Representative fluorescence images for Figure 4E. Scale bar: 50 µm.
